# Heavy alcohol drinking downregulates ALDH2 gene expression but heavy smoking up-regulates SOD2 gene expression in head and neck squamous cell carcinoma

**DOI:** 10.1186/s12957-017-1225-1

**Published:** 2017-08-25

**Authors:** Dong Jin Lee, Hyung Min Lee, Jin Hwan Kim, II Seok Park, Young Soo Rho

**Affiliations:** Department of Otolrayngology-Head and Neck Surgery, Kangnam Sacred Heart Hospital, Hallym University College of Medicine, Seoul, South Korea

**Keywords:** Microarray, ALDH2, SOD2, Expression, Alcohol, Smoking, Survival, HNSCC

## Abstract

**Background:**

This study aims to determine the relationship between expression levels of ALDH2 and SOD2 genes and clinical parameters such as alcohol drinking, tobacco smoking, primary site of HNSCC, and human papilloma virus (HPV) state.

**Methods:**

Gene expression data were obtained from gene expression omnibus (GEO accession number: GSE65858). Clinical data (*N* = 270) including survival result, gender, age, TNM stage, primary site of HNSCC, HPV status, alcohol drinking, and tobacco smoking habit were analyzed according to gene expression pattern.

**Results:**

ALDH2 gene was expressed in low levels in patients with heavy alcohol consumption. It was expressed in high (*p* = 0.01) levels in patients with no or light alcohol consumption. ALDH2 gene was also expressed in low levels in patients with oral cavity cancers or hypopharynx cancers. However, ALDH2 gene was expressed in high (*p* = 0.03) levels in patients with oropharyngeal cancers or laryngeal cancers. HPV-positive patients were found to have high (*p* = 0.02) expression levels of ALDH2. SOD2 gene was expressed in high (*p* = 0.005) levels in patients who had greater mean pack-year of tobacco smoking. Based on log rank test, the group of patients with high expression of ALDH2 showed better (*p* = 0.002) clinical results than those with low expression of ALDH2. Difference of survival results between ALDH2 high-expressed group and ALDH2 low-expressed group was validated in another cohort (GSE39368, *N* = 138).

**Conclusions:**

Heavy alcohol drinking downregulates ALDH2 gene expression level. Heavy smoking up-regulates SOD2 gene expression level in patients with head and neck squamous cell carcinoma. The group of patients with low expression levels of ALDH2 showed significantly poorer survival results compared to those with high expression levels of ALDH2.

## Background

Head and neck squamous cell carcinoma (HNSCC), the fifth most common type of cancer, accounts for 600,000 newly diagnosed cancer cases with 300,000 deaths worldwide every year [1]. Despite novel surgical and pharmaceutical treatment methods developed for HNSCC, its survival rate has not been improved over the last two decades [2]. It is widely accepted that HNSCC is strongly associated with alcohol drinking and tobacco smoking [3]. It has been estimated that 75% of HNSCC cases in the USA are caused by alcohol drinking and tobacco smoking [4].

In general, the metabolism of ethanol, the major component of alcoholic beverages, consists of two steps [5]. First, alcohol dehydrogenase (ADH) enzyme converts ethanol to acetaldehyde. Second, aldehyde dehydrogenase (ALDH) converts acetaldehyde to acetate. Acetaldehyde is a toxic material that has been established as a strong carcinogen [6]. The second oxidation step is largely dependent on ALDH2 enzyme encoded by ALDH2 gene.

As for smoking, tobacco smoke produces a variety of free radicals, reactive oxygen species (ROS), and reactive nitrogen species (RNS). They will produce superoxide, hydrogen peroxide, and nitric oxide which can cause oxidative damage and oxidative stress to cells [7]. Superoxide dismutases (SODs) are enzymes that can catalyze the dismutation of superoxide into oxygen or hydrogen peroxide [8]. There are three distinctive SODs: copper/zinc SOD (Cu/ZnSOD or SOD1), manganese SOD (MnSOD or SOD2), and extracellular SOD (ecSOD or SOD3, and Cu/ZnSOD) [9]. Among them, SOD2 is localized at the mitochondria. It acts as an endogenous antioxidant enzyme that limits the oxidative burden [10]. SOD2 can convert superoxide into hydrogen peroxide and oxygen in the mitochondria. It plays a major role in protecting cells from oxidative damage [11].

A number of studies have demonstrated the correlation between ALDH2 gene or SOD2 gene polymorphisms and susceptibility to HNSCC [12–17]. However, the expression pattern of these two genes in HNSCC patients in terms of the amount of alcohol drinking and tobacco smoking has not been reported.

Therefore, the objectives of this study were to analyze the expression pattern of ALDH2 and SOD2 genes using available microarray data (GSE65858 and GSE39368) [18] and determine the relationship between the expression level of these two genes and clinical parameters such as alcohol drinking, tobacco smoking, primary site of HNSCC, and human papilloma virus (HPV) state.

## Methods

### Gene expression data and clinical information

Gene expression data (mRNA-seq) and clinical data of 270 head and neck squamous cell carcinomas were obtained from gene expression omnibus (GEO) (http://www.ncbi.nlm.nih.gov/geo/, accession number:GSE65858) [18]. Clinical data included survival data, gender, age, TNM stage, primary site and HPV status, alcohol, and smoking habit. Amount of alcohol consumption is divided into four groups: non, light 1–30 g/day, moderate 31–60 g/day, heavy > 60 g/day (Table 1).

**Table 1:** Statistical analysis of clinical variables according to ALDH2 and SOD2 expression level

		ALDH2 high	ALDH2 low	*p* value	SOD2 high	SOD2 low	*p* value
Age	Mean	60.3	59.8	0.74	60.3	59.7	0.65
Gender	Male	123	100	0.50	135	88	0.44
	Female	29	18		25	22	
Smoking	Mean pack-year	29.4	32.6	0.28	34.3	25.9	*0.005
Alcohol amount	Heavy	44	48	*0.01	16	15	0.15
	Moderate	30	33		43	41	
	Light	57	27		41	22	
	None	21	10		60	32	
Stage	I–II	35	20	0.28	36	19	0.37
	III–IV	117	98		124	91	
Primary site	Oral cavity	37	46	*0.03	47	36	0.94
	Oropharynx	65	37		62	40	
	Larynx	30	18		29	19	
	Hypopharynx	16	17		20	13	
HPV	Positive	42	18	*0.02	34	26	0.77
	Negative	109	100		125	84	

*means statistically significant *p* value

### Gene selection and unsupervised clustering

We selected ADH1B, ADH1C, and ALDH2 as alcohol-related genes and SOD1, SOD2, and SOD3 as smoking-related genes. The BRB-ArrayTools software program (http://linus.nci.nih.gov/BRB-ArrayTools.html) was used to analyze gene expression data [19]. After gene expression data were gene-median centered, we performed unsupervised clustering of these three genes in 270 HNSCCs cases. A heatmap was generated using the Cluster and TreeView software programs [20].

### Statistical analysis

All statistical analyses were performed in the R language (http://www.r-project.org).

Comparing mean age between groups was performed using paired *t*-test. Qui-square test were performed for all other statistical analysis. The *p* value < 0.05 was considered as a significant difference.

### Survival analysis

The association of each group with overall survival was evaluated using Kaplan-Meier plots and log rank test. Overall survival was defined as the time from surgery to death. Data were censored when a patient was alive without recurrence at last follow-up. The *p* value < 0.05 was considered as a significant difference.

### Significant canonical signaling pathways enriched in each group

Pathway analysis was carried by using Ingenuity Pathways Analysis (Ingenuity, Redwood City, CA), and genes from the dataset that were associated with a canonical pathway in the Ingenuity Pathways Knowledge Base were considered for the analysis.

### Validation in another cohort

We applied this finding to another validation cohort (GSE39368, *N* = 138) to show the robustness of this survival difference between ALDH2 high-expressed group and ALDH2 low-expressed group.

## Results

### Unsupervised clustering and boxplot

**Fig. 1:**
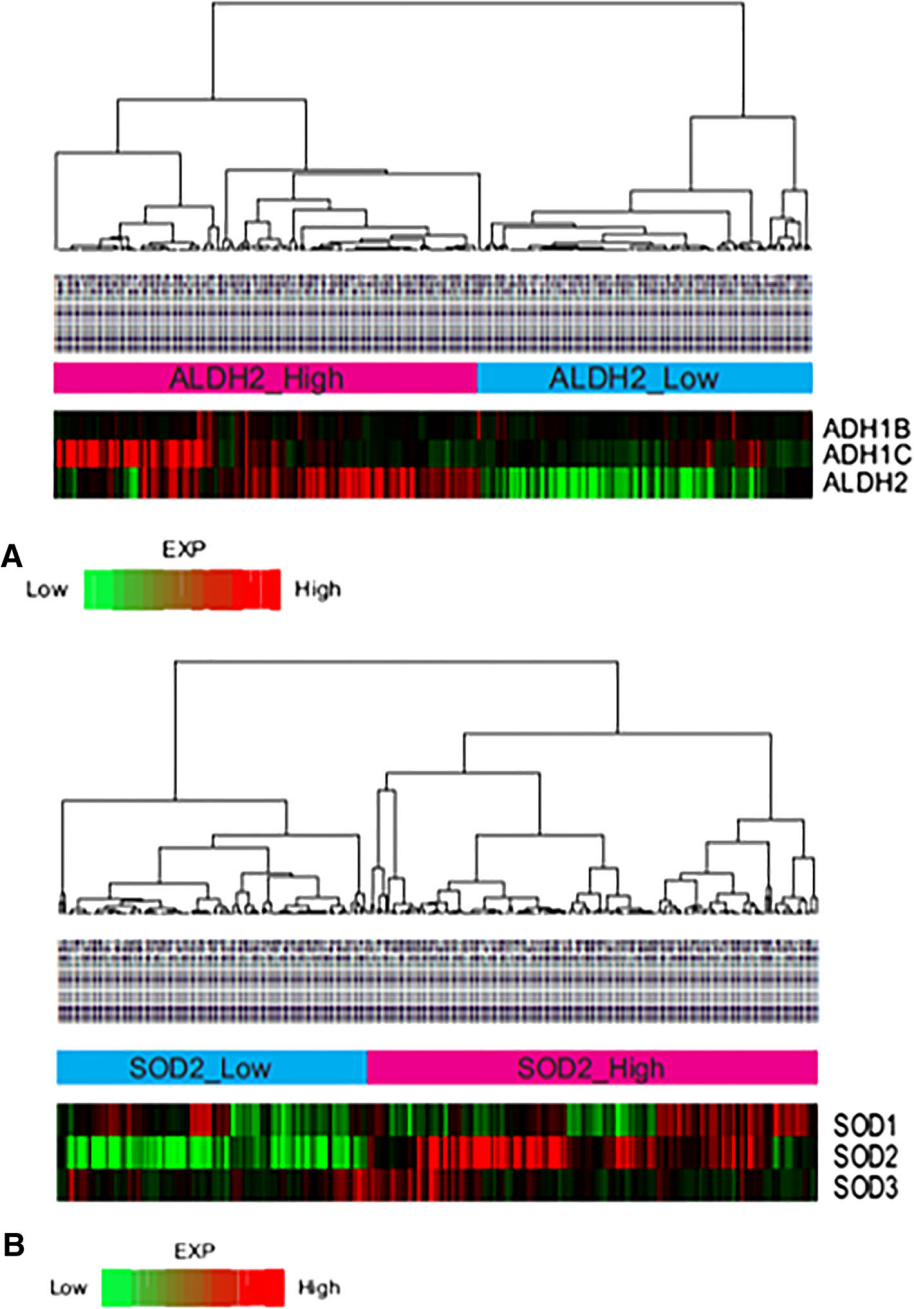
Unsupervised clustering of 270 cases HNSCCs in GSE65858 according to aldehyde dehydrogenase (ALDH) and superoxide dismutase (SOD) genes reveals 2 molecular subtypes–ALDH2_expression_High vs ALDH2_expression_Low (**a**), and SOD2_expression_High vs SOD2_expression_Low (**b**). The data are given in matrix format. The color *red* or *green* in cell reflects relative high or low expression levels, respectively, as indicated in *scale bar*

**Fig. 2:**
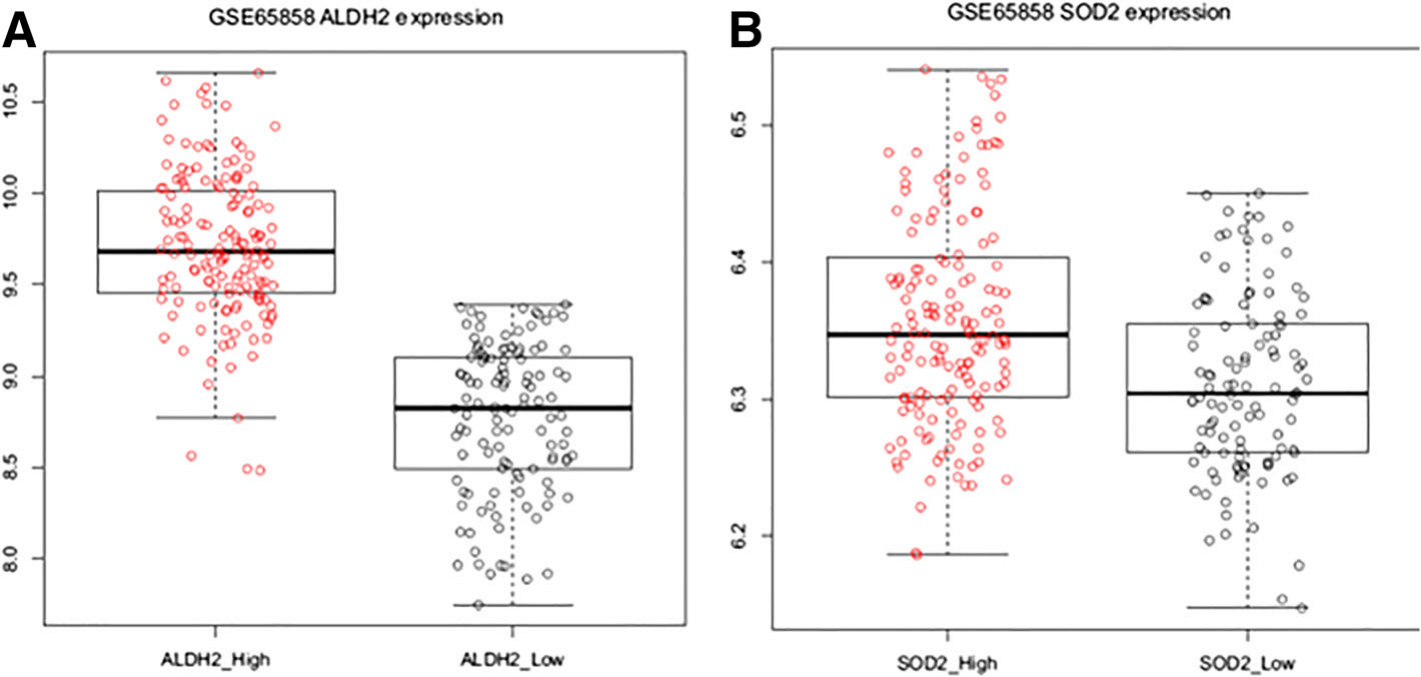
Boxplots shows the difference of gene expression level between two groups. **a** The difference of ALDH2 gene expression level. **b** The difference of SOD2 gene expression level

We performed hierarchical clustering in an unsupervised and unbiased manner using cluster 3.0. We found two expression subtypes in alcohol-related genes and smoking-related genes (Fig. 1). For ALDH2 genes, two groups (ALDH2 high-expressed group vs. ALDH2 low-expressed group) were obtained (Fig. 1a). For SOD2 genes, two groups (SOD2 high-expressed group vs. SOD2 low-expressed group) were also obtained (Fig. 1b). Compared to ALDH2 and SOD2 genes, other genes were not divided into two groups (Fig. 1). The difference in expression level between the two groups was determined by boxplot analysis (Fig. 2).

### Statistical analysis for clinical variables

Results of statistical analysis for clinical variables including age, gender, smoking state, alcohol habit, stage, primary site, and HPV state of 270 HNSCC cases are summarized in Table 1. ALDH2 gene was expressed at low levels in patients with heavy alcohol consumption. However, it was expressed at high levels in patients with no or light alcohol consumption. ALDH2 gene was also expressed at low levels in patients with oral cavity cancers or hypopharynx cancers. However, it was expressed at high levels in patients with oropharyngeal cancers or laryngeal cancers (Table 1). HPV positive patients were found to have high expression levels of ALDH2. They were found in the ALDH2 high-expressed group. In contrast, SOD2 gene was expressed at high levels in patients who had greater mean pack-year of tobacco smoking. There was no statistically significant difference in other variables between the SOD2 high-expression and the SOD2 low-expression group (Table 1).

### Survival analysis

**Fig. 3:**
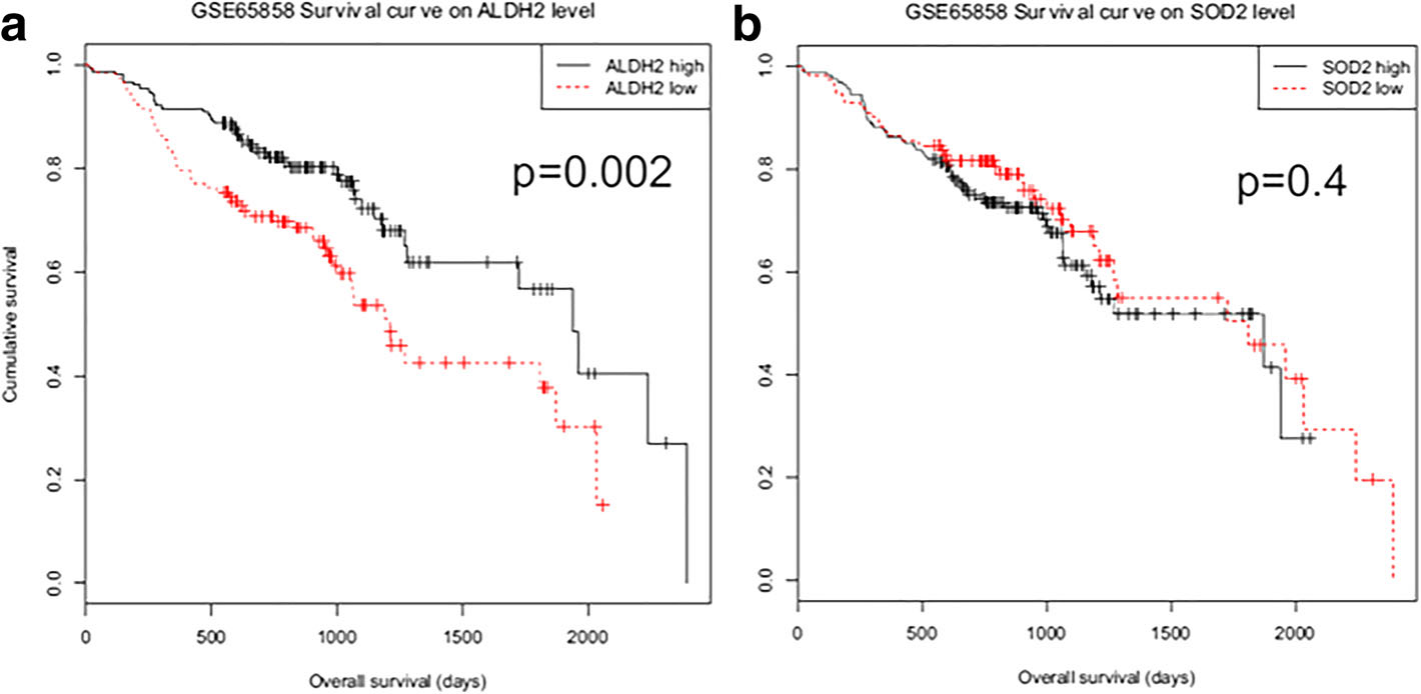
Kaplan-Meier survival curve according to ALDH2 and SOD2 expression level. ALDH2_high group showed better prognosis when compared with ALDH2_low group (*p* = 0.002, (**a**)). In contrast, SOD2 gene expression level did not show any survival difference (*p* = 0.4, (**b**))

When we compared the KM survival curve and log rank test for each high- and low-expressed group, ALDH2 high-expression group showed better survival results compared to the ALDH2 low-expression group (Fig. 3a). However, the survival results of SOD2 high-expression group were not significantly different from those of SOD2 low-expression group (Fig. 3b).

### Significant canonical signaling pathways enriched in each group

Pathway analysis revealed that ALDH2 high-expression group was enriched in canonical pathways such as Cdc42 signaling, lymphotoxin β receptor signaling, RhoA signaling, and intrinsic prothrombin activation pathway (Table 2). SOD2 high-expression group was also enriched in canonical pathways such as TREM1 signaling, PPAR signaling, PI3K/AKT signaling, and protein kinase A signaling pathway (Table 2).

**Table 2:** Altered canonical pathways in each group

	-Log (*p* value)	Ratio	z-score	Molecules
Altered pathways in ALDH2 high-expressed group				
Role of NFAT in regulation of the immune response	4.82E00	2.34E−01	3.157	BLNK,CD247,RAF1,HLA-DOA,CD3E,CD4,GNA11,FCER1A,HLA-DQA1
Cdc42 signaling	3E00	2.04E−01	−2.828	CD247,RAF1,HLA-DOA,MAP3K11,CD3E,ARPC1B,HLA-DQA1,CDC42EP2
Lymphotoxin β receptor signaling	1.32E00	2.04E−01	2.714	IKBKB,MAP3K14,VCAM1,AKT1,CASP9,LTA,PIK3CG,LTB,IKBKE,PIK3CB
RhoA signaling	4.37E−01	1.31E−01	−2.309	NEDD4,CFL1,NRP2,ARPC1B,ANLN,GNA12,RTKN
Intrinsic prothrombin activation pathway	5.98E−01	1.72E−01	−2.236	COL1A2,COL1A1,COL5A3,COL10A1,COL3A1
Altered pathways in SOD2 high-expressed group				
TREM1 signaling	8.12E00	2.27E−01	3.638	RELA,CXCL8,TREM1,NLRP3,PLCG1,IL6,CCL3,NOD2,CCL2,CASP1
PPAR signaling	3.95E00	1.4E−01	−3.606	RELA,IL36G,IL1A,NRAS,RRAS2,NFKBIA,HSP90AB1,PPARD,IL1B
PI3K/AKT signaling	3.78E00	1.22E−01	2.324	TSC1,RELA,NRAS,YWHAH,PIK3R1,MAP3K5,ITGA3,NFKBIA,RRAS2
Protein kinase A signaling	3.16E00	7.77E−02	−2.294	RELA,YWHAH,PDE7A,PTPN2,ANAPC10,PTPN12,GNG7,PHKA2,GNB3
TNFR1 signaling	1.83E00	1.22E−01	2.236	RELA,NFKBIA,TNFAIP3,NFKBIB,TNF,CASP7
IL-1 signaling	3.45E00	1.32E−01	2.121	RELA,IL1A,GNB3,NFKBIA,GNA15,GNB2,ADCY6,IRAK3,NFKBIB,GNG5

### Validation in another cohort

**Fig. 4:**
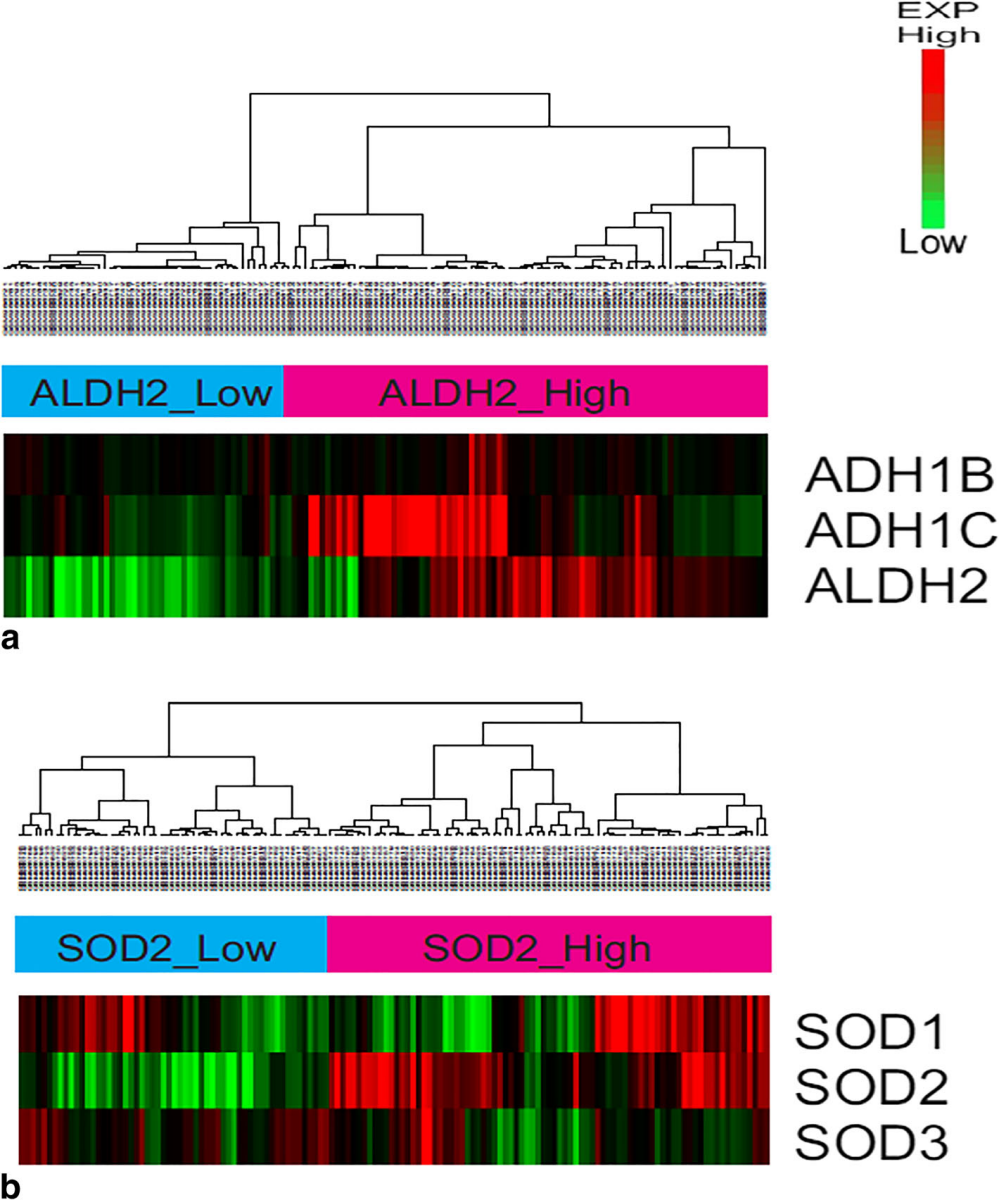
Unsupervised clustering of 183 cases HNSCCs in GSE39368 according to aldehyde dehydrogenase (ALDH) and superoxide dismutase (SOD) genes reveals two molecular subtypes–ALDH2_expression_High vs ALDH2_expression_Low (**a**), and SOD2_expression_High vs SOD2_expression_Low (**b**)

**Fig. 5:**
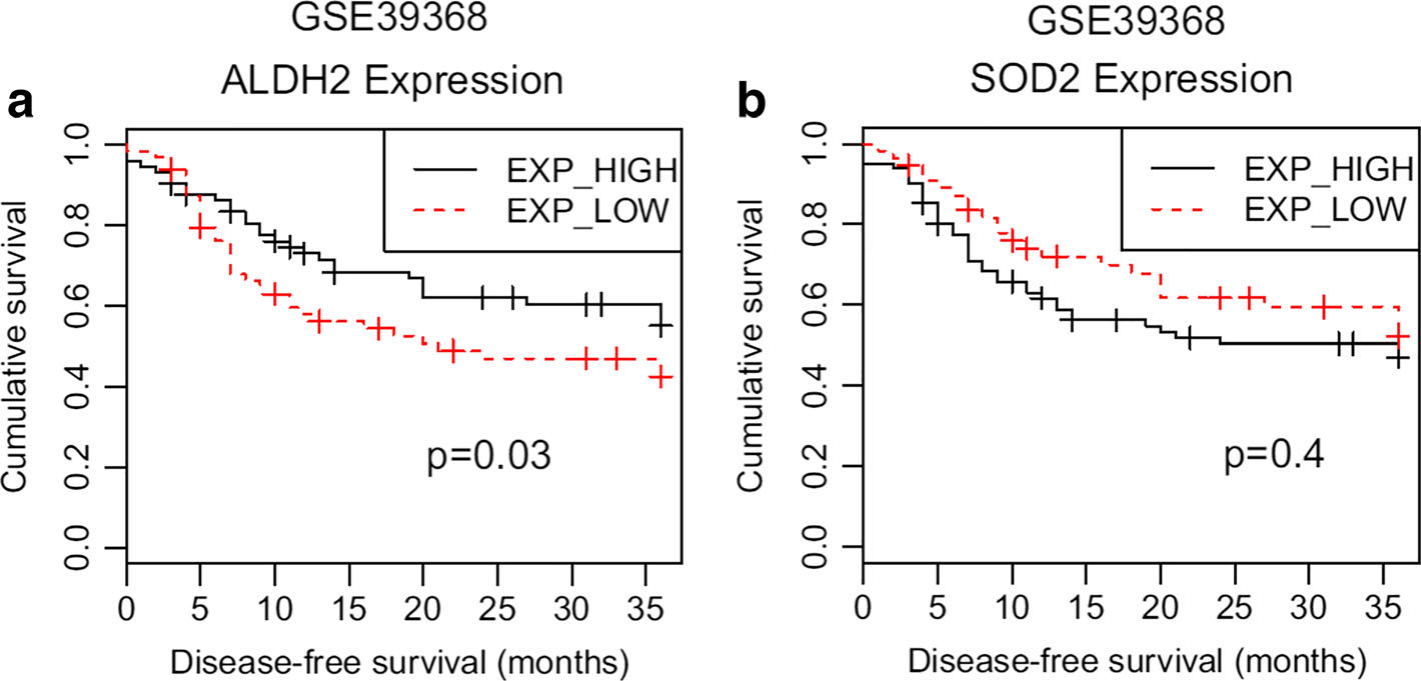
Kaplan-Meier survival curve according to ALDH2 and SOD2 expression level in validation cohort (GSE39368). ALDH2_high group showed better prognosis when compared with ALDH2_low group (*p* = 0.03, (**a**)). In contrast, SOD2 gene expression level did not show any survival difference (*p* = 0.4, (**b**))

We applied this finding to another validation cohort (GSE39368, *N* = 138) to show the robustness of this survival difference between ALDH2 high-expressed group and ALDH2 low-expressed group. For ALDH2 genes, two groups (ALDH2 high-expressed group vs. ALDH2 low-expressed group) were obtained (Fig. 4a). For SOD2 genes, two groups (SOD2 high-expressed group vs. SOD2 low-expressed group) were also obtained (Fig. 4b). When we compared the KM survival curve and log rank test for each high- and low-expressed group, ALDH2 high-expression group showed better survival results compared to the ALDH2 low-expression group (Fig. 5a, *p* = 0.03). However, the survival results of SOD2 high-expression group were not significantly different from those of SOD2 low-expression group (Fig. 5b, *p* = 0.4).

## Discussion

In this study, we evaluated the association between expression levels of alcohol metabolism genes or ROS metabolism genes and the amount of alcohol drinking or tobacco smoking. We also determined the association of gene expression patterns with clinical variables such as primary site, HPV state, stage, and survival result. Among alcohol metabolism genes, ALDH2 gene expression pattern divided the microarray data into two groups: ALDH2 high-expression group (*N* = 152) and ALDH2 low-expression group (*N* = 118) (Fig. 1a). Interestingly, heavy alcohol consumption group showed low expression levels of ALDH2 with poor overall survival results (Table 1 and Fig. 3a). Microarray data (*N* = 270) were also divided into two groups by SOD2 expression pattern (Fig. 1b). However, in contrast to the relationship between ALDH2 expression and alcohol consumption amount, heavy smoking group showed high SOD2 gene expression (Table 1). Interestingly, there was no correlation between SOD2 expression level and survival results (Fig. 3b).

Alcohol drinking and tobacco smoking are significant risk factors for head and neck cancer [3]. In general, alcohol metabolism involves two steps. First, alcohol is converted into acetaldehyde by ADH. Second, acetaldehyde is oxidized into nontoxic acetate by ALDH [5]. Among all classes of ADH and ALDH isoenzymes, ADH1B, ADH1C, and ALDH2 are the main ethanol-metabolizing enzymes [21, 22]. Therefore, we used these three genes to analyze the expression pattern of alcohol metabolism genes. As for ROS metabolism genes, SODs are enzymes that catalyze the removal of superoxide free radicals [8]. SODs are present in all aerobic living cells. This is probably because O_2_^−^ is a common product of oxygen metabolic reactions. In mammals, there are three distinctive SODs: SOD1 (Cu/ZnSOD), SOD2 (MnSOD), and SOD3 (extracellular SOD). SOD1 is the major intracellular form of SODs. It is primarily cytosolic [23]. In contrast, SOD2 is exclusively localized in the mitochondrial matrix (MM) [23], while SOD3 is the secreted form mainly associated with the extracellular matrix of different tissues [24]. In this study, we used these three superoxide dismutases genes to analyze the expression patterns of ROS metabolism genes. Among these three alcohol-related genes, only ALDH2 gene showed distinguished expression pattern. Similarly, among the three smoking-related genes, only SOD2 gene showed distinguished expression subtype.

Several studies have demonstrated the correlation between ALDH2 gene or SOD2 gene polymorphisms and susceptibility to cancer development. Zhao et al. have reported that ALDH2 Glu504Lys SNP is a potential candidate genetic risk factor for a variety of chronic diseases such as cardiovascular disease, cancer, and late-onset Alzheimer’s disease [12]. Chung et al. have suggested that minor alleles of ADH1B (rs1229984) and ALDH2 (rs671) not only are associated with the risk of upper aerodigestive tract cancer, but also can potentiate the carcinogenic effects of alcohol [13]. Hidaka et al. have reported that ALDH2 AA allele carriers who drink > 150 g/week have an increased risk of cancer development compared to GG genotype carriers who drink 0 to < 150 g/week [15]. As for SOD2, Han et al. have reported that polymorphism of SOD2 T5482C might be closely associated with increased susceptibility to the development and differentiation of gastric cancer in Korean population [16]. All these SNP studies have emphasized the important roles of ALDH2 and SOD2 in alcohol drinking and smoking-induced cancer development. However, up to date, no study has reported the expression levels of these genes, especially using microarray mRNA-Seq data. In this study, we used mRNA-seq data and evaluated the relationship between expression levels of ALDH2 and SOD2 genes and the amount of alcohol drinking or smoking consumption.

In statistical analysis, the amount of alcohol consumption, primary site, and HPV state showed statistically significant difference between ALDH2 high-expression group and ALDH2 low-expression group (Table 1). Patients with heavy alcohol consumption showed low ALDH2 expression levels, while patients with no or light alcohol consumption showed high ALDH2 expression levels (Table 1). There are two possible hypotheses to explain such results. First, cumulative acetaldehyde itself might have downregulated the expression of ALDH2 gene directly or indirectly using other upstream or downstream molecules. Second, the liver functions of heavy alcoholics usually are not so good. Their anabolic functions such as mRNA synthesis and protein synthesis are usually decreased. The finding that patients in the heavy smoking group showed high SOD2 expression in this study supports the second hypothesis.

In this study, we used Ingenuity Pathway Analysis (IPA) to investigate up-regulated and downregulated pathways in ALDH2 high-expression group and SOD2 high-expression group. Intrinsic prothrombin activation pathway is up-regulated in ALDH2 low-expression group (*z*-score 2.236, Table 2). Garcia-Banuelos et al. have investigated the relationship between ALDH2 SNP and liver cirrhosis [25]. Heavy alcohol drinking can cause liver damage and activate intrinsic prothrombin activation pathway, especially in the ALDH2 low-expression group. Chao et al. have reported the interplay between superoxide dismutase genes and PPAR signaling [26]. In our study, the SOD2 high-expression group showed decreased PPAR signaling pathway (Table 2).

This study has some limitations. First, this study is a retrospective study. Second, no data about liver function state of patients enrolled in this study were available. Comparing liver functions between heavy alcoholics and those with no or light alcohol consumption is needed as we mentioned above. A prospective and functional study is needed to provide more precise information about the role and prognostic aspect of ALDH2 and SOD2 genes in head and neck cancer.

## Conclusions

In conclusion, this is the first study describing the relationship between the amount of alcohol drinking or smoking consumption and alcohol or ROS metabolizing gene expression level. In this study, heavy alcohol drinking downregulated ALDH2 gene expression while heavy smoking up-regulated SOD2 gene expression in head and neck squamous cell carcinoma patients (*N* = 270). This result can be used to predict the survival results of HNSCC patients, especially those heavy alcoholics with HNSCC.
